# The Placarding of Private Houses

**Published:** 1898-10

**Authors:** 


### The Placarding of Private Houses.

As regards the relation of Boards of Health to the profession, the position taken by Dr. Meigs in a recent paper on this subject, seems an eminently proper one. He states:

"There are several reasons why houses should not be placarded and various ways in which placarding is productive of harm. The state of panic created in the public mind is certainly an evil, for it is perfectly well known how difficult it is to deal with frightened people. Probably the greatest harm which comes from the enforcement of placarding is that it greatly increases the number of cases that are concealed. The public and physicians joins hands in this practice and it is only what might be expected. Respectable physicians have told me that they never report cases of diphtheria if they can avoid it, or report them only when they think death is imminent and the risk of detection becomes very great.

The placarding of houses for contagious disease involves trampling under foot the rights of individuals and such violent intrusion upon family privacy that we ought to pause and ask if we are not in danger of losing one of our precious heritages derived from our English origin, that "every man's house is his castle. How much immunity from disease have we obtained in return? The mortality records would seem to indicate that we are less free from typhoid fever and diphtheria in Philadelphia than they are in London where yellow placards are never used, but where there is a good sanitary system."

The views here advanced will at once receive the endorsement of the majority of our profession, for not a few of its members have had unpleasant experiences in matters pertaining to the enforcement of sanitary laws and have already reached the conclusion that an enterprising Board of Health, which happens to possess little knowledge and less tact, is capable of inflicting a great amount of damage not only upon the attending physician, but the community as well. As the law now stands, the physician in attendance upon a contagious case has to do only with its medicinal treatment. The authorities, however, have the right to invade the premises, isolate the patient and recommend to the family a course of disinfection which best accords with their own ideas, but which may or may not meet the approval of the medical attendant. The pernicious activity of health boards has already worked much injury to the medical profession and has forced it to resent undue interference and the desire to assume the sanitary control of infectious cases, which are being skillfully provided for. As the author of the paper truly states:

"Our health authorities should direct their efforts to cleanliness and measures to improve the general hygiene of the city instead of advertising by the posting of yellow placards the existence of disease after it has come. The right method is for the physician to notify and the health officers to prevent."—*New England Medical Monthly*.

